# Diagnosis of Dehydrated Hereditary Stomatocytosis in a 60-Year-Old Female Patient

**DOI:** 10.7759/cureus.89808

**Published:** 2025-08-11

**Authors:** Kevin Han, Kafi Thomas, Leen Khoury, Jordonna Brown

**Affiliations:** 1 Internal Medicine, State University of New York Downstate Health Sciences University, Brooklyn, USA; 2 Hematology and Oncology, State University of New York Downstate Health Sciences University, Brooklyn, USA; 3 Hematology and Oncology, State University of New York Downstate Medical Center, Brooklyn, USA

**Keywords:** adulthood, dehydrated hereditary stomatocytosis, hemolytic anemia, hereditary xerocytosis, next generation sequencing (ngs), piezo1 mutation

## Abstract

Dehydrated hereditary stomatocytosis (DHS), also known as hereditary xerocytosis, is a rare autosomal dominant hemolytic anemia characterized by mild to moderate, rarely severe, hemolysis as a result of decreased red blood cell (RBC) osmotic fragility leading to RBC dehydration. Patients with DHS are usually diagnosed at birth, during childhood, or in early adulthood. We herein present a case of DHS diagnosed in a 60-year-old female patient with no previously known history of a blood disorder; numerous stomatocytes were seen on peripheral smear, with next-generation sequencing (NGS) revealing a heterozygous PIEZO1 gene mutation.

## Introduction

Dehydrated hereditary stomatocytosis (DHS) was first reported in three young siblings of Swiss German ancestry in 1971 with the unique characteristics of stomatocytosis, decreased osmotic fragility, auto-hemolysis, and abnormal intracellular electrolyte balance [1]. The exact nature of the defect was not discovered till 2012, when two missense mutations at the FAM38A gene encoding PIEZO1 protein were found to be associated with DHS [2]. The term “PIEZO,” originally from the Greek word “píesi,” meaning pressure, is a mechanoreceptor of RBC membranes and functions as a biological pressure sensor that regulates the cell volume [3]. Further studies suggest that the increased PIEZO1 channel activity by gain-of-function mutation in DHS leads to an increase in the influx of sodium ions and efflux of potassium ions with subsequent net water loss [4], thereby directly contributing to red blood cell (RBC) dehydration, shrinkage, and hemolysis.

In addition to clinical symptoms related to anemia, DHS can also present variably with jaundice, cholelithiasis, splenomegaly, and incidental findings such as hemosiderosis of organs like the liver. Splenectomy is contraindicated in DHS due to the increased risk of thrombosis [5, 6]. The degree of anemia depends on the magnitude of hemolysis and the robustness of erythropoiesis, but it is generally mild. One retrospective case series of 126 patients shows an average hemoglobin (Hb) of 13.1 g/dl [7]. Rarely, DHS is a part of a pleiotropic syndrome, along with pseudohyperkalemia and pre-/perinatal edema [8], which can be severe, causing fetal hydrops [9]. Though inciting events like exercise have been reported to trigger decompensated hemolysis [10], patients with DHS usually have partial to full compensation of the hemolysis. The treatment for DHS is supportive care with favorable outcomes in most patients.

## Case presentation

A 60-year-old Afro-Caribbean female patient presented to the emergency department (ED) complaining of one to two weeks of generalized weakness, headaches, and shortness of breath on exertion. She denied vaginal or rectal bleeding, a history of blood disorders or prior transfusions, and reported no family history of hemolytic anemia, only sickle cell trait in her son. Labs revealed Hb 6.4 g/dL with mean corpuscular volume (MCV) 105.8 fl. Her baseline hemoglobin (Hb) was 11 g/dL around nine months prior to presentation. Physical examination was notable for conjunctival pallor. The fecal occult blood test was negative. She was transfused with two units of packed RBCs with an increase in Hb to 7.8 g/dL and an improvement in her symptoms (Table 1). She was discharged with a plan for follow-up with gastroenterology for endoscopic evaluation, given a recent positive *Helicobacter pylori* (*H. pylori*) test.

**Table 1:** Labs on presentation and post-transfusion hemoglobin results

Test name	Results	Reference range	Units
Hemoglobin (Hb)	6.4	12-16	g/dL
Mean cell volume (MCV)	105.8	78-95	fL
Post-transfusion hemoglobin (Hb)	7.8	12-16	g/dL

She returned to the ED six days later with recurrent symptoms of anemia, namely fatigue, worsening exercise tolerance, and dizziness. Upon physical exam, the patient presented with left upper abdominal intermittent pain as well as pruritus. Labs showed Hb of 6.2 g/dl; MCV 96.3 fl; mean corpuscular hemoglobin concentration (MCHC) 31.8 g/dl; haptoglobin <10 mg/dl; lactate dehydrogenase (LDH) 996 U/L; reticulocyte% 7.76%; total bilirubin 2.4 mg/dl; direct bilirubin 1.9 mg/dl; ferritin 2052 ng/ml; serum iron 53 ug/dl; and total iron binding capacity (TIBC) 282 ug/dl (Table 2).

**Table 2:** Labs on re-presentation to the hospital

Test name	Results	Reference range	Units
Hemoglobin (Hb)	6.2	12-16	g/dL
Mean cell volume (MCV)	96.3	78-95	fL
Mean corpuscular hemoglobin concentration (MCHC)	31.8	30.5-35.5	g/dL
Haptoglobin	<10	30-200	mg/dL
Lactate dehydrogenase (LDH)	996	135-214	U/L
Reticulocyte%	7.76	0.5-2.5	%
Total bilirubin	2.4	0.0-1.2	mg/dL
Direct bilirubin	1.9	0.0-0.3	mg/dL
Ferritin	2052	13-150	ng/mL
Iron	53	37-145	ug/dL
Total iron binding capacity (TIBC)	282	250-410	ug/dL

**Figure 1:**
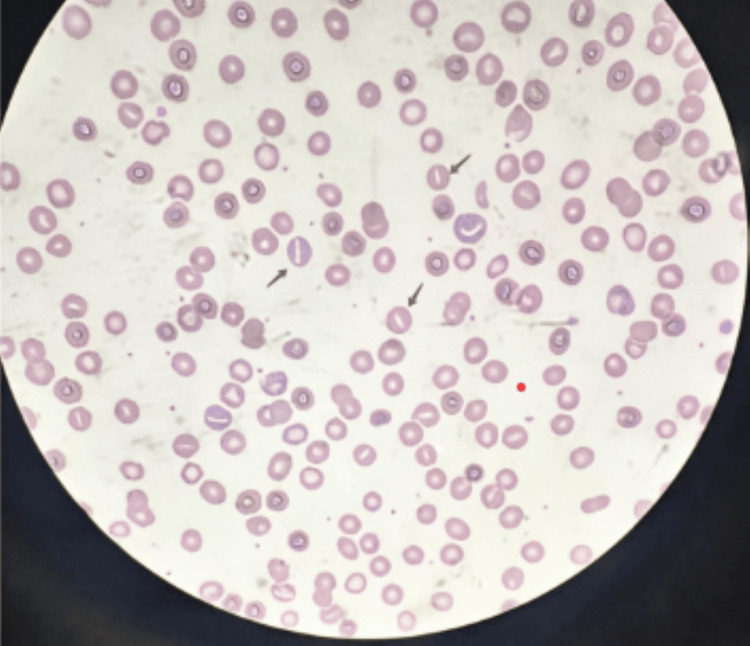
Peripheral blood smear displaying black arrows pointing at stomatocytes (Wright-Giemsa stain, 100x).

**Figure 2:**
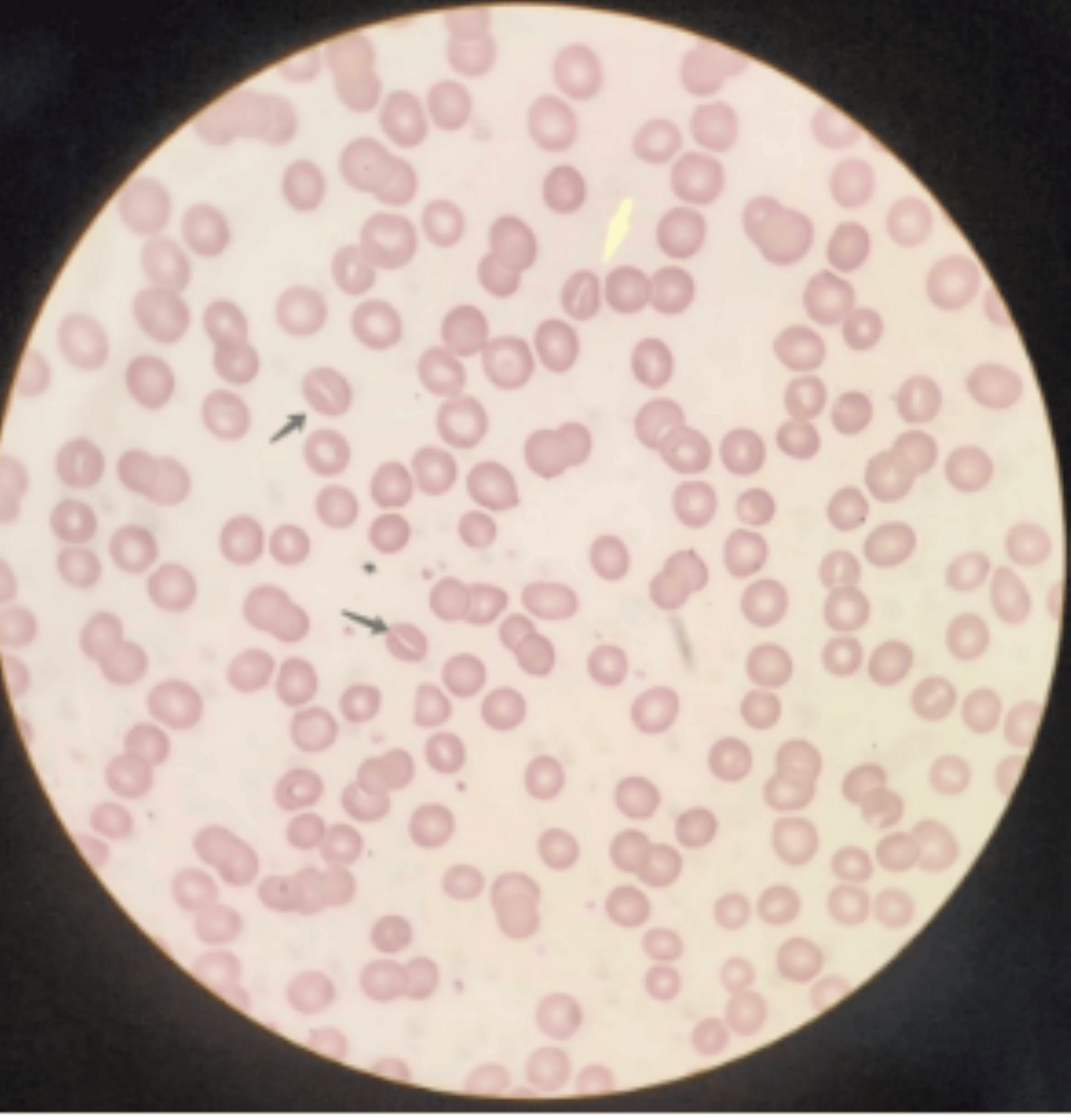
Peripheral blood smear; yellow and black arrows point at stomatocytes (Wright-Giemsa stain, 100x).

Magnetic resonance imaging (MRI) of the abdomen showed multiple focal splenic infarcts with splenomegaly and findings suggestive of secondary hemosiderosis in the liver. Peripheral smear showed polychromasia and numerous stomatocytes, with no fragmented red blood cells or nucleated red blood cells (Figures 1, 2). Further laboratory studies showed decreased RBC osmotic fragility but negative results for direct antiglobulin test (DAT), cold agglutinin titer, genetic testing for hemochromatosis, and flow cytometry for paroxysmal nocturnal hemoglobinuria (PNH). Delayed hemolytic transfusion reaction was excluded, given a negative DAT. She received a total of three units of packed RBCs and was otherwise managed supportively and discharged home. 

She was seen in the hematology clinic one month after discharge. She reported no further symptoms of anemia. Her Hb was back to her baseline of 11.9 g/dL. Given the patient’s presentation and lab evidence of non-immune hemolytic anemia (DAT negative), the presence of numerous stomatocytes on peripheral smear, decreased RBC osmotic fragility, elevated ferritin not related to blood transfusions, and the absence of hereditary hemochromatosis and splenic infarcts, the suspicion for DHS was raised. RBC membrane sequencing using next-generation sequencing (NGS) was later performed and revealed a heterozygous PIEZO1 gene mutation, supporting the diagnosis of dehydrated hereditary stomatocytosis. The patient was started on folic acid for chronic hemolysis, and a T2* MRI was requested to assess for iron deposition in the heart, but it was not available at our center. Her Hb has remained stable between 11 and 12 g/dL since then, with no evidence of hemolysis. Chelation therapy was considered for her hyperferritinemia, but her ferritin levels significantly improved with the resolution of the hemolysis.

## Discussion

The classic estimated prevalence of DHS has been suggested to be around 1:50,000 based on clinical presentation [6]. Recent studies derived from large U.S. laboratory databases indicate a possible six-fold higher prevalence of 1:8000 after reviewing nearly 20% of the US population and selecting the hereditary xerocytosis (HX) candidates,” reflecting that a great proportion of patients with this condition could potentially remain underdiagnosed [11]. Moreover, this condition is also often misdiagnosed as other hemolytic anemias, for example, other congenital membrane defect disorders like hereditary spherocytosis (HS) or enzymopathies like glucose-6-phosphate dehydrogenase deficiency (G6PD), given overlapping clinical and hematologic features.

The anemia associated with DHS is generally mild and does not immediately raise concerns for an underlying blood disorder. Therefore, the diagnosis of DHS in adult patients requires a high index of suspicion. Clinical clues to the diagnosis of DHS include elevated MCV, MCHC, mean corpuscular hemoglobin (MCH), ferritin, and transferrin saturation (TSAT) in the presence of stomatocytes on peripheral blood smear (although present in less than 20%) and decreased osmotic fragility. The osmotic fragility test helps to differentiate DHS from other hemolytic anemias by assessing the resistance of RBCs to hemolysis in hypotonic solutions. In DHS, RBCs have increased membrane surface area and low total cation content, making them more resistant to osmotic stress, resulting in decreased osmotic fragility, whereas other hemolytic anemias, such as HS, are associated with increased osmotic fragility. It is notable that the patient's MCHC in this case report was normal for reasons that are unclear. 

Further evaluation with the osmotic gradient ektacytometry test is useful in confirming the diagnosis. Ektacytometry directly assesses cell hydration and can therefore identify all types of hereditary stomatocytosis based on distinct deformability profiles [12]. Ektacytometry could not be performed in this case due to the very limited laboratories in the US that do this testing. 

The use of NGS is widely adopted and has become a cornerstone for diagnosing cases of DHS [13]. Apart from the PIEZO1 gene, another novel gene mutation, KCNN4, has been found to be associated with DHS [14] and can be identified on NGS. In our case, KCNN4 was tested and reported negative.

Unlike HS, for which splenectomy can be beneficial, DHS patients are at a higher risk for thromboembolic events post splenectomy; therefore, splenectomy should be avoided [15]. The exact mechanism has not been well established, but studies have indicated a possible relation with abnormal erythrocyte endothelial adherence [16]. Patients who, unfortunately, had splenectomies should be evaluated for lifelong anticoagulation [17]. Treatment for DHS is supportive, with blood transfusions as needed.

Patients with DHS with hyperferritinemia should be regularly followed up with iron studies and potential MRI imaging to assess for cardiac and hepatic iron deposition, and iron chelation should be considered in cases of iron overload. In our case, patient ferritin levels were monitored at three-month intervals, demonstrating a gradual normalization over time. Additionally, studies have suggested iron overload or hepatosiderosis could be the very first findings in mild cases [18].

## Conclusions

Our case highlights that DHS is a rare cause of non-immune hemolysis that often requires a high index of suspicion, especially when diagnosed in late adulthood. Accurate diagnosis necessitates a comprehensive approach involving clinical assessment, laboratory and biochemical evaluation, and genetic testing. This case also underscores the utility of NGS in identifying causative mutations such as those in PIEZO1. Reassuringly, most cases of DHS are mild and have a limited impact on the patient’s quality of life. Conservative management, with regular laboratory monitoring and avoidance of splenectomy, remains the cornerstone of care.
